# The Coronavirus Disease 2019 (COVID-19): Key Emphasis on Melatonin Safety and Therapeutic Efficacy

**DOI:** 10.3390/antiox10071152

**Published:** 2021-07-20

**Authors:** Eva Ramos, Francisco López-Muñoz, Emilio Gil-Martín, Javier Egea, Iris Álvarez-Merz, Sakshi Painuli, Prabhakar Semwal, Natália Martins, Jesús M. Hernández-Guijo, Alejandro Romero

**Affiliations:** 1 Department of Pharmacology and Toxicology, Faculty of Veterinary Medicine, Complutense University of Madrid, 28040 Madrid, Spain; eva.ramos@ucm.es; 2 Faculty of Health Sciences, University Camilo José Cela, C/Castillo de Alarcón 49, Villanueva de la Cañada, 28692 Madrid, Spain; flopez@ucjc.edu; 3 Neuropsychopharmacology Unit, Hospital 12 de Octubre Research Institute (i + 12), Avda. Córdoba, s/n, 28041 Madrid, Spain; 4 Portucalense Institute of Neuropsychology and Cognitive and Behavioural Neurosciences (INPP), Portucalense University, R. Dr. António Bernardino de Almeida 541, 4200-072 Porto, Portugal; 5 Thematic Network for Cooperative Health Research (RETICS), Addictive Disorders Network, Health Institute Carlos III, MICINN and FEDER, 28029 Madrid, Spain; 6 Nutrition, Food & Plant Science Group NF1, Department of Biochemistry, Genetics and Immunology, Faculty of Biology, University of Vigo, 36310 Vigo, Spain; egil@uvigo.es; 7 Health Research Institute, Hospital Universitario de la Princesa, 28006 Madrid, Spain; javier.egea@inv.uam.es; 8 Molecular Neuroinflammation and Neuronal Plasticity Research Laboratory, Hospital Universitario Santa Cristina, Instituto de Investigación Sanitaria-Hospital Universitario de la Princesa, 28006 Madrid, Spain; 9 Department of Pharmacology and Therapeutic, Teófilo Hernando Institute, Faculty of Medicine, Universidad Autónoma de Madrid, Av. Arzobispo Morcillo 4, 28029 Madrid, Spain; iris.alvarez@estudiante.uam.es (I.Á.-M.); jesusmiguel.hernandez@uam.es (J.M.H.-G.); 10 Ramón y Cajal Institute for Health Research (IRYCIS), Hospital Ramón y Cajal, Carretera de Colmenar Viejo, Km. 9100, 28029 Madrid, Spain; 11 Department of Biotechnology, Graphic Era University, Dehradun, Uttarakhand 248002, India; sakshipainuli@geu.ac.in (S.P.); semwal.prabhakar@geu.ac.in (P.S.); 12 Uttarakhand State Council for Science and Technology, Dehradun, Uttarakhand 248007, India; 13 Faculty of Medicine, Institute for Research and Innovation in Health (i3S), University of Porto, Alameda Prof. Hernâni Monteiro, 4200-319 Porto, Portugal; ncmartins@med.up.pt; 14 Institute for Research and Advanced Training in Health Sciences and Technologies, Cooperativa de Ensino Superior Politécnico e Universitário (CESPU), Rua Central de Gandra, 1317, 4585-116 Gandra, Portugal

**Keywords:** melatonin, SARS-CoV-2, safety, adjuvant therapy, mitochondria, inflammation, antioxidant, circadian rhythms, clinical trials

## Abstract

Viral infections constitute a tectonic convulsion in the normophysiology of the hosts. The current coronavirus disease 2019 (COVID-19) pandemic is not an exception, and therefore the severe acute respiratory syndrome coronavirus 2 (SARS-CoV-2) infection, like any other invading microbe, enacts a generalized immune response once the virus contacts the body. Melatonin is a systemic dealer that does not overlook any homeostasis disturbance, which consequently brings into play its cooperative triad, antioxidant, anti-inflammatory, and immune-stimulant backbone, to stop the infective cycle of SARS-CoV-2 or any other endogenous or exogenous threat. In COVID-19, the corporal propagation of SARS-CoV-2 involves an exacerbated oxidative activity and therefore the overproduction of great amounts of reactive oxygen and nitrogen species (RONS). The endorsement of melatonin as a possible protective agent against the current pandemic is indirectly supported by its widely demonstrated beneficial role in preclinical and clinical studies of other respiratory diseases. In addition, focusing the therapeutic action on strengthening the host protection responses in critical phases of the infective cycle makes it likely that multi-tasking melatonin will provide multi-protection, maintaining its efficacy against the virus variants that are already emerging and will emerge as long as SARS-CoV-2 continues to circulate among us.

## 1. Introduction

Recently, the rapid spread of new coronaviruses throughout the world has had a great impact on worldwide societal and economic development, besides it is being considered the most crucial global health disaster of the century [1]. The ongoing pandemic of coronavirus disease 2019 (COVID-19) caused by the severe acute respiratory syndrome coronavirus 2 (SARC-CoV-2) has a plethora of presentations, drifting from asymptomatic to a severe state of the disease, with a wide variety of symptoms [2]. Globally, considering its high mortality and unexpected rapid spread, several groups of academia, industry, and the government sector are working together to develop an effective and safe treatment against COVID-19. Besides pharmacological therapy, few scientific groups are working on non-pharmacological molecules that may reduce the risk of getting infected with COVID-19.

Melatonin plays an important role in the regulation of different physiological functions including free radical detoxification and immune system enhancing, besides exerting direct anti-inflammatory, anti-cancer, neuroprotective, cardiovascular, and anti-diabetic effects [3,4,5,6,7,8]. At a physiological level, the synthesis of melatonin takes place during darkness in a light–dark cycle; therefore, it is described as the indoleamine of darkness and/or a chronobiotic molecule [9].

In the last months, moved by impotence in the face of the horror caused by COVID-19, numerous researchers have emphasized the therapeutic potential of melatonin [10,11,12]. The pineal indoleamine is one of the molecules about which probably more pages have been written on its evolutionary origin, functionality, and applicability in clinical settings. As a result, its functional biology is one of the best-known, and we now realize that it goes far beyond the traditional role in controlling the circadian and seasonal cycles. On the contrary, melatonin is currently considered a universal cell protector with signaling actions in a myriad of biochemical processes essential for cell and tissue homeostasis [13,14]. In this sense, among the plethora of mediations that melatonin carries out, the regulation of immunoinflammatory responses and the protection against oxidative damage are the focus of interest. These two properties are the main pillars on which the commitment of many scientists to the administration of melatonin for the combat of SARS-CoV-2′s pathogenicity [15] and other virus types [16,17,18].

The vast research around SARS-CoV-2 developed last year signals a double indirect contribution of melatonin-mediated responses to the natural defense against COVID-19 morbimortality [19]. First, the viral “superimmunity” of bats [20] and the presumed primary reservoir of SARS-CoV-2 and other corona/filoviruses, along with the decline of melatonin in the elderly [21], allow us to hypothesize that (i) high endogenous melatonin provides greater antiviral resistance to bats than to humans and (ii) higher melatonin in childhood protects children from COVID-19 better than adults [22,23]. Consistent with this assumption, it makes sense that in silico drug repurposing projections from human/SARS-CoV-2 global protein–protein interactome network and retrospective and observational analyses of COVID-19 and non-COVID-19 cohorts have revealed the therapeutic as well as the antiviral prophylactic mechanistically based utility of melatonin, alone or in combination with other compounds [24,25]. Additionally, it is equally conceivable that from strictly theoretical considerations based on the electronic structure of the molecule, the functionality of melatonin has been predicted, and docking simulations have suggested the potential efficacy of the indoleamine in the treatment of COVID-19 [26], presuming its role as SARS-CoV-2 protease inhibitor [27]. Furthermore, to address potential treatments, network-based prediction and a propensity score-matching observational study of more than 18,000 COVID-19 patients were combined and showed melatonin reducing the capacity to drop COVID-19 risk by 64% [25]. On the empirical side, the observational study of a large COVID-19 cohort showed that patients taking melatonin had a reduced risk of SARS-CoV-2 infection, even compared with those treated with potent angiotensin-converting enzyme inhibitors or angiotensin II receptor blockers, among others [25]. Likewise, individuals on melatonin treatment, along with other pharmacological, genre, ethnicity, and age features, have been positively discriminated as reduced-risk for COVID-19 by a logistic regression algorithm over more than 11,000 people [28]. Moreover, the translational applicability of this molecule stands out over other therapy drug candidates to effectively and safely curtail the COVID-19 pandemic [10,25,29], as well as to enhance the immune response to vaccination [16,30,31,32]. 

Secondly, another plane in favor of melatonin in managing the immunopathology burden of COVID-19 is directly envisaged from the predictable efficacy of its wide-ranging capacities. This is the case of aberrant “cytokine storm syndromes”, critical events of massive production and release of pro-inflammatory mediators orchestrated by the abnormal response of the immune-inflammatory system and which determine the fatal course of COVID-19 patients [33]. The prolonged recruitment in the lower respiratory tract of the host innate inflammatory lineages (macrophages, neutrophils, and mast cells) promote epithelial/endothelial apoptosis, diffuse alveolar epithelium damage, and tissue remodeling, leading to acute respiratory distress syndrome (ARDS), hypercoagulability, and clotting microthrombi, as well as switching to aerobic glycolysis responsible for the lactate dehydrogenase elevation and lactic acidosis frequently diagnosed in COVID-19 patients [34,35]. Later, hyper-inflammation by SARS-CoV-2 eventually leads to severe failure of the lungs, heart, and other peripheral organs, septic shock, and the irreversible perturbation of those whole biosystems that configure COVID-19 pathogenesis and determines the prognosis of patients, often ending in death [36,37]. However, at the same time, this immune-inflammatory status provides clues for the design of therapeutic approaches capable of adequately modulating the immune response. In this regard, melatonin can modulate innate and adaptive immunities, avoiding overreaction and the pathological escalation of the cytokine storm. In other words, the indoleamine helps to enhance host tolerance and overcome the viral cycle, preventing the systemic damage of tissues and organs [18,38]. From the biological perspective, melatonin raises barriers against virus invasion and replication, so the infective strategy usually involves torpedoing its synthesis and signaling to hinder the crucial antioxidant, metabolic, and immune-modulatory actions played by the indoleamine in the defense of cellular homeostasis [19,39,40]. In accordance with the above, as melatonin is produced endogenously and is also present in our diet, many researchers propose to supplement natural intakes with the indoleamine to emulate in COVID-19 patients the benefits observed in entities with phenotypic similarities. The high tolerability of melatonin, even at high doses [41,42], enhances the plausibility of efficient and safe melatonin-based COVID-19 treatments.

The alliance of dysfunctional innate/adaptive immunities with inflammation explains the enhanced susceptibility and devastating progress of COVID-19 in the elderly and patients affected by chronic primary diseases [43,44]. Only the increased risk of the high SARS-CoV-2 infectivity rate with age represents an immense public health challenge, since a potential population of more than 1.7 billion people worldwide of all countries and ethnicities may end up suffering from severe COVID-19 [45]. In anticipation of future scenarios of equal or greater complexity than COVID-19, the reinforcement of the host defense mechanisms provided by melatonin has the additional interest that a similar base treatment could be effective for other upcoming viral or microbial nosological entities associated with exacerbated immunoinflammation [18]. In sum, it is an ancient molecule at the forefront of future therapeutics. This is the promise that the urgent program of systematic investigation, which we want to claim herein, may put in our hands.

## 2. SARS-CoV-2: From Biological Features to Pathophysiology

Coronaviruses are the largest group of viruses responsible for respiratory, gastrointestinal, and central nervous system (CNS) infections [46]. They can be divided into four genera, including alpha (α), beta (β), gamma (Υ), and delta (δ). Among these, alpha and beta only infect mammals, whereas gamma and delta primarily infect birds with few deviations [47]. Based on its phylogenic analysis and genomic construction, the SARS-CoV-2 has its place in the beta group (Hu et al., 2020). Taxonomically, SARS-CoV-2 belongs to the family Coronaviridae, order Nidovirales and genus Coronavirus. Morphologically, they are large, enveloped, positive-sense, single-stranded, RNA viruses, with a genome size of ~30,000 bases [48], typically round or multi-shaped with a 65–125 nm diameter consisting of a triple Spike glycoprotein. With high throughput sequencing, research groups have done the sequencing of SARS-CoV-2 and declared that the RNA includes 14 putative open reading frames (ORFs) encoding 27 proteins [49,50]. Moreover, it was confirmed that the sequence of SARS-CoV-2 has similarities with SARS-CoV (40%) and MERS-CoV (40%) [51]. Coronavirus genomes generally encode four additional structural proteins, including the Spike proteins (1128–1472 amino acids), membrane protein (218–263 amino acids), envelope protein (74–109 amino acids), and nucleo-capsid protein (349–470 amino acids) [52] The N protein holds the RNA genome, and S, E, and M proteins together create the viral envelope [50]. Apart from this, these proteins may be involved in the entry of viral particles into the host cell after binding with host angiotensin-converting enzyme 2 (ACE-2) [53], promoting virulence, virus morphogenesis, assembly, budding, and folding of genomic RNA into the nucleo-capsid [54,55]. ACE-2 is a functional receptor hijacked by SARS-CoV-2 for cell entry, similar to SARS-CoV [56,57]. Briefly, it is a type 1 membrane protein expressed in the lungs, heart, kidney, and intestine mainly associated with cardiovascular diseases [58], and these human organs are vulnerable to SARS-CoV-2 [59]. Xiao and co-workers [60] report on the multiplication of virus in the mucosal epithelium of upper and lower respiratory tract and GI mucosa, while in other cases, patients present with heart injury, kidney failure, acute liver, and diarrhea, as non-respiratory symptoms [37,61,62]. Regarding respiratory symptoms, fever, coughing, and shortness of breath are the common symptoms of SARS-CoV-2 (https://www.cdc.gov/coronavirus/2019-ncov/symptoms-testing/symptoms.html, Accessed date 22 February 2021), but olfactory and gustative disorders have also been reported in infected patients [63]. More recently, Wang and co-workers reported in a metanalysis that hypertension, diabetes, chronic obstructive pulmonary disease (COPD), and cardiovascular and cerebrovascular diseases are significant risk factors for COVID-19-related mortality [64].

Regarding SARS-CoV-2 transmission, it is recognized as predominantly occurring through respiratory droplets, direct contact, and potential fecal–oral route transmission. In addition, it was recently underlined that SARS-CoV-2 can survive outside the body for a long time. For example, van Doremalen and co-workers [65] reported on the viability and stability of SARS-CoV-2 on different surfaces and found that the plastic and stainless-steel surfaces were more stable for SARS-CoV-2 than copper and cardboard, with the viable virus being detected for up to 72 h on these surfaces.

## 3. Melatonin and Health: A Brief Overview

Endogenous melatonin was proposed to be a potential key factor in promoting human health in the last century [66]. Indeed, describing its antioxidant properties raises the idea of supplementing with melatonin to protect brain health in the advanced aged population [66]. In the last two decades, numerous researchers have investigated the plethora of melatonin’s actions protecting and regulating many aspects in humans [67]. There is, apparently, a lack of serious adverse effects after its supplementation [68]. Notwithstanding, a particular population should be monitored to clarify this relatively safe profile. Melatonin´s pleiotropy, safety profile, and inexpensive cost have led researchers to investigate in humans the promising results elicited preclinically [69,70,71]. 

Mainly, among the most significant actions of supplemented melatonin described to date, we must highlight its antioxidant, free-radical scavenging, immunomodulatory, and anti-inflammatory activity, which makes melatonin a powerful tool in the treatment of any disease that involves one or more of these components. There is an increasing interest in melatonin against cancer; recent studies report a very promising oncostatic profile of melatonin in several types of cancer and associated effects that seem to enhance chemotherapy [72,73,74,75,76].

To reach a clinical therapeutic approach to all the pathologies that melatonin seems to improve, alone or in combination with other treatments, more economic support is needed. As melatonin is not patentable, it will probably not result in a high profit to companies; therefore, the clue may be to invest more public money, which will revert to better health in the population. Reportedly, melatonin seems to promote patient health and moreover save the lives of more critical patients.

## 4. Melatonin Effects in COVID-19: A Clinic-Critical Perspective

### 4.1. Ongoing Clinical Trials on Melatonin Effects in SARS-CoV-2 Infection

As mentioned above, melatonin is an endogenous non-patentable molecule; thus, clinical trials do not expect to obtain great economic benefits; indeed, most of the clinical trials developed are public-funded [69]. To date, there is a relevant lack of clinical trials concerning melatonin´s therapeutic effects versus SARS-CoV-2’s directly or indirectly related pathologies [77,78]. Clinical trials are mandatory to confirm the preclinical efficacy and safety observed [79]. Notwithstanding, the COVID-19 pandemic has launched several clinical trials to test doses, administration routes, and efficacy against SARS-CoV-2.

To our knowledge, 11 clinical trials are currently ongoing, registered in the Cochrane Library (ISSN 1465–1858) collection of databases (12 February 2021). Table 1 depicts a summary of the main characteristics of the ongoing trials. Only one clinical trial evaluates melatonin as a protective tool versus COVID-19; a daily dose of 2 mg of melatonin is orally administered for 12 weeks to test whether melatonin may protect healthcare workers compared to the placebo group [80,81]. Interestingly, there is only one study on whether intravenous melatonin, administered to patients located in the Intensive Care Unit (ICU), can reduce mortality [81,82]. The other published protocol evaluates the efficacy and safety of 50 mg of melatonin daily administered during seven days, compared to the standard therapeutic regimen [83]. As shown in Table 1, the rest of the trials test oral melatonin efficacy in mild or moderate patients; noteworthily, the administered doses go from 3 to 100 mg. As we previously pointed, selecting the correct doses is absolutely necessary to obtain reliable results [18,84]. Regarding the ongoing trials, very different profiles of melatonin’s therapeutic value are being examined, from prevention to reducing mortality. These differences would probably make more difficult the overall analysis, whereas they may give a broad therapeutic profile. There are no results published to date; we are looking forward to analyzing and comparing results related to doses and administration routes, contributing to understanding the required doses and efficacy of melatonin against COVID-19.

### 4.2. Sleep and Circadian Rhythms Dysregulation and COVID-19

Interference with the pleiotropic beneficial actions of melatonin, particularly the inhibition of the secretory nocturnal peak by excessive lighting at dark time, the so-called “light-at-night idea” or LAN hypothesis [85], is potentially health-harmful and a risk factor for multiple disorders [86]. The promising profile of melatonin to treat COVID-19 is based on hindering several key steps for SARS-CoV-2 infectivity and the onset of disease, as well as on the improvement of respiratory and non-respiratory clinical features and the resynchronization of circadian disruption commonly found in affected individuals [10,87]. Specifically, nocturnal pineal melatonin is synchronized with an acute proliferation of hematopoietic stem and progenitor cells [88], and, accordingly, sleep deprivation led to disorders associated with chronic inflammatory tone. Therefore, we consider: (i) that the aged population represents the most vulnerable group to COVID-19 infection; (ii) the natural age-related decline of melatonin levels, (iii) that higher exposure of individuals to LAN also reduces melatonin levels and disrupts circadian rhythmicity. Therefore, if nighttime illumination is shielded from the patients in the intensive care units (ICU), it preserves higher levels of circulating melatonin [89] and stimulates immunity in COVID-19 patients [90] and (iv) stress factors involved in the disruption of the sleep pattern such as depression, work overload, and long confinements with time watching TV and surfing the internet. Moreover, a recent report has postulated an association between increased physiological risk of COVID-19 infection and prolonged night-shift work [91]. Given this evidence, restitution of a regular circadian melatonin pattern would permit the protection of the most vulnerable workers under chronic light pollution. Therefore, we must optimize an adequate chronotherapeutic treatment following the body’s circadian rhythms to improve the sequels, not only of COVID-19, but also of the consequences in the context of the pandemic situation [87]. In this regard, the chronobiotic agent melatonin regulates the sleep/wake cycle and other circadian rhythms and, even at high doses (50–100 mg p.o.), improves and prolongs the chronobiotic effect [87]. In fact, during the pandemic time, insomnia symptoms affected about 60% of the Italian population, and following the recommendations of five scientific societies, the melatonin prolonged release at the dose of 2 mg was chosen as the pharmacological option in subjects ≥55 years old over 13 weeks [92].

Delirium and sleep disturbances such as confusion, somnolence, and stupor were reported in almost 15% of hospitalized COVID-19 patients [93]. In the case of delirium, many factors trigger it; dysbalanced neurotransmitters, proinflammatory cytokines, tissue hypoxia, and sleep deprivation. In this sense, the administration of melatonin up to 10 mg has been recommended in hyperactive delirium associated with COVID-19 infection in the ICU [94]. In this regard, between the considerations in the treatment of hyperactive or mixed COVID-19-associated ICU delirium, a high dose of the melatonin (10–15 mg enteric at night) was used to regulate the sleep–wake cycle and assists in the treatment of delirium in combination with Suvorexant [95].

The presence of comorbidities in COVID-19 patients such as diabetes, cardiovascular, or chronic kidney diseases can increase the risk of developing the severe disease [96]. Thus, individuals with diabetes present a decrease in melatonin production associated with high circulating glucose levels [97]. Therefore, when they are infected by SARS-CoV-2 must be important that maintain a good “sleep hygiene” to regulate their melatonin and glucose levels [98]. Interestingly, since melatonin may have beneficial effects for sleep disorders on COVID-19 outcomes, the obstructive sleep apnea could be another research field to examine its possible mechanistic pathways and its relationship with the effect of the COVID-19 pandemic [99]. Recently, it was also hypothesized that melatonin deficiency may predispose those autism spectrum disorder patients who have low melatonin output to contract COVID-19 [100].

### 4.3. SARS-CoV-2 and Melatonin Target Proteins: CD147, DPP4, AhR, PAK1, and EGFR

Refractory hypoxemia and myocardial injury are physiopathological conditions exhibited in COVID-19 patients under mechanical ventilation for the treatment of acute respiratory distress syndrome (ARDS), which is caused by SARS-CoV-2-induced acute oxidative erythrocytic damage. In this respect, erythrocytes are targeted by SARS-CoV-2 spike (S) protein through binding to CD147 receptors, which are widely expressed in many cell types such as hematopoietic and endothelial cells, leukocytes, and platelets. Furthermore, CD147 possesses a functional role in facilitating SARS-CoV infection, and it has been postulated as the primary SARS-CoV-2 receptor in a novel route of invasion [101]. Subsequently, disruption of Ca^2+^ homeostasis and alteration in the CD147-cyclophilin A signaling pathway may contribute to thrombotic events and inflammatory injury in the lung and heart failure [102]. Therefore, targeting CD147 to reduce inflammation and severity of the disease progression could be an interesting strategy to be addressed. In this context, melatonin has been used successfully to reduce cardiac damage through the blockade of the CD147 signaling pathway [103] and it may play an important role in diabetic COVID-19 patients [104]. Another proposed door for SARS-CoV-2 infection is the highly lung-expressed DiPeptidyl Peptidase-4 (DPP4) receptor, which has been found negatively regulated by melatonin in mice and in in vitro models of calcific aortic valve disease [105], suggesting the putative regulation of SARS-CoV-2 entry by the indolamine [106]. In this regard, the aryl hydrocarbon receptor (AhR) has attracted attention due to its multiple roles in the co-coordination of cellular internalization and pathophysiology associated with the infective process of SARS-CoV-2, particularly the “third cytokine storm” caused by induction of the indoleamine 2,3-dioxygenase (IDO)/kynurenine/AhR pathway [106]. AhR is activated by the discharge of inflammatory cytokines during the initial hyper-inflammatory phase that suppresses the endogenous immune response and facilitates the completion of the viral infective cycle, as reported in mouse hepatitis virus-infected cells [107]. Specifically, AhR activation stimulates gut permeability and microbiome dysbiosis [108], which increases TLRs’ activators and reduces the production of the short-chain fatty acid, butyrate [109]. Butyrate and melatonin enhance mitochondrial acetyl-CoA and OXPHOS while acetyl-CoA coactivates the mitochondrial synthesis of melatonin. Thus, AhR activation increases gut permeability/dysbiosis and reduces gut butyrate and thereby the availability of mitochondrial melatonin, particularly in immune cells [106]. The reciprocal antagonism between AhR and melatonergic system is endorsed by AhR activation following viral invasion and the subsequent exhaustion of melatonin availability [77] as well as by the common occurrence of activated AhR and decreased pineal melatonin in comorbidities at high risk of fatal COVID-19 such as respiratory, cardiovascular, and metabolic disorders. It is therefore conceivable that melatonin administration may inhibit AhR and in this way drive part of its anti-inflammatory and immune-enhancing potential. Interestingly, regarding COVID-19, several nutraceuticals are functional antagonists of AhR and thereby their food intake has been postulated as a potential modulator of COVID-19 severity [106,110]. Then, the question that arises is: could melatonin be a relevant part of natural compounds capable of moderating the course of the disease in critically ill patients? The functional biology of the indoleamine and some preclinical data [27,29,80,81,82,111,112,113] support an affirmative answer.

Molecular modeling tools facilitate theoretical prediction in the search for therapeutic targets that are vulnerable to SARS-CoV-2. Among them, chymotrypsin-like protease (Mpro) would be an interesting target to inhibit virus replication. It has been observed that the interaction between melatonin and Mpro, based on the docking energy results, highlights the therapeutic potential of melatonin by binding to viral protease Mpro [27]. In this same line, a detailed quantum-mechanical research predicted the capacity of melatonin to dock with SARS-CoV-2 proteins [26]. This interesting fact makes urgent further studies to incorporate melatonin in the COVID-19 treatment regime. Additionally, another interesting target enzyme to counteract the SARS-CoV-2 viability and progression is the major “pathogenic” kinase PAK1, whose malfunction induces inflammation, viral infection, and immuno-suppression; therefore, PAK1-blockers are considered to have an anti-coronaviral effect. Recently, melatonin has been related to an anti-PAK1 activity, which would evidence, one more time, the multifunctionality displayed by this indolamine [114].

Early observations have emphasized the epidermal growth factor receptor (EGFR) as a direct target in antiviral therapy; more precisely, EGFR signaling inhibition can prevent SARS-CoV-2 replication in the infected cells [115], and melatonin would be able to modulate growth factor receptor signaling activity inhibiting SARS-CoV-2 replication [116], mitigating the development of severe clinical symptomatology.

### 4.4. Melatonin Impact from an Immune–Metabolic Perspective

Numerous retrospective studies have shown that suffering from certain primary pre-existing pathologies facilitates symptomatic COVID-19 and increases the baseline of mortality [117,118,119,120,121]. In the middle of 2020, the Centers for Disease Control and Prevention announced that among COVID-19 patients, cardiac disorders and type 2 diabetes were the two most common predisposing conditions, with a participation of each of them around 1/3 of diagnoses [122]. In addition, the need for hospitalization as well as the clinical course and death risk among comorbid patients were significantly poor compared with those without these primary pathologies. Indeed, from this perspective, chronic metabolic disease (obesity, type 2 diabetes and metabolic syndrome) as well as ischemic/non-ischemic cardiovascular and cerebrovascular pathology, along with chronic lung injury, are the most prevalent comorbidities that speed up the onset of COVID-19 and the worsening of the patients, having up to ≈10-fold higher risk of death [123]. Abundant concordant data on the facilitation of multifactorial COVID-19 by these cardiometabolic disorders common in the elderly have been incessantly collected worldwide through the direct screening of large COVID-19 cohorts and longitudinal retrospective studies [117,124]. 

The resulting evidence of observational and clinical studies points out that all these closely related predisposing entities configure a pathological cluster involving the alteration of metabolic profile, the dysregulation of the innate immune reactivity, and low-grade chronic inflammation [125], which facilitates the cytokine storms and associated organ derangements. In this regard, the fact that inflammation and disturbances of energy metabolism can impair the immune response [57] had led to scrutinizing the immunological implications of obesity and the release of inflammatory factors by obese adipose tissue [126]. Specifically, the chronic low-grade inflammation associated with obesity was demonstrated as a conditional risk for metabolic disorders because macrophages recruited into adipose tissue result activated until their pro-inflammatory M1 phenotype, then releasing inflammatory mediators into the local and systemic environment [127]. On the other hand, cross interactions between innate immunity and metabolism facilitated the crystallization of “immunometabolism” [128], in whose conceptual framework the immune–metabolic interface is seen as a bidirectional control system [129]. Furthermore, immune response encompasses inflammatory activation, and for this reason, both processes must be approached as a unit of action. This integrative vision sheds light on the observation that metabolic and/or immunoinflammatory disruptions may contribute to prolonging hospitalization and/or to increasing lethality among COVID-19 patients [130]. Likewise, another relational consideration to annotate in the natural history of the current pandemic is the involvement of mitochondrial dysregulation in the onset of the pathological phenotype of COVID-19 and its primary predisposing disorders [131,132]. SARS-CoV-2 impairs the mitochondrial-associated antiviral signaling system (MAVS) which allows the virus to evade the surveillance of the host’s innate immune system [132], as summarized in Section 4.6. In accordance with the latter, infections due to immunodeficiency are common in inherited mitochondrial disorders [45], clearly highlighting the pronounced effects that mitochondrial failure has on immune and inflammatory responses.

Low-grade basal inflammation and weakening of immune and antioxidant defenses are omnipresent in the elderly (inflammaging), metabolic syndrome abnormalities (metaflammation), and the spectrum of other pre-existing disorders behind the proclivity to COVID-19 onset and aggravation. At the same time, the aging and chronification of nosological entities that coexist with COVID-19, particularly those exhibiting insulin resistance, are accompanied by a progressive fall in circulating melatonin [132,133,134]. It is noteworthy that clinical trials and studies with animal models indicate that supplementation with melatonin may be a valid strategy to tackle symptomatology and halt the progression of COVID-19 [134,135,136], perhaps because the indoleamine helps to counteract the debilitating phenotype of aging [137] and predisposing pathologies [133,138,139]. In this regard, based on the orchestration of endocrine, paracrine, and autocrine responses, melatonin may be considered a smart controller of the innate and adaptive immunity [140] and inflammation [141], a neuroimmune-endocrine regulator [142] that collaborates with the maintenance of the internal environment and the mitigation of cell/tissue perturbations. Consequently, the homeostatic mediation of melatonin must be understood as a cell protector endowed with multiple restorative capacities of metabolic and immunoinflammatory disturbances and with particular potential utility in respiratory and lung viral infections [142]. Recently, mitochondria have revealed themselves as the core target of melatonin activity [143,144], and hence, normalization of mitochondrial function and reduction of nitro-oxidative stress are the backbone of pleiotropic action performed by the indoleamine therein [145]. Indeed, melatonin protects mitochondria against glycolytic energy metabolism instilled by SARS-CoV-2 [146]. In this way, indoleamine reduces oxidative stress and contributes to preventing hyper-inflammation and the innate immune exacerbation that led to the feared storm of cytokines. Furthermore, the return to OXPHOS replenishes the mitochondrial pool of acetyl-CoA, which is needed as co-substrate for the in-situ synthesis of the indoleamine, thus closing the virus-dependent disruption of mitochondrial melatonin. Consistent with all of the above, in different experimental settings and clinical trials, melatonin has shown remarkable capacities in the normalization or attenuation of insulin resistance, inflammation, nitro-oxidative stress, and chronodisruption of peripheral oscillators [134,141]. In addition, melatonin signaling pathways have an extensive influence on glucose homeostasis and energy metabolism [147]. Correspondingly, the administration of complementary melatonin has demonstrated successful outcomes in the management of diabetes and metabolic syndrome [148,149,150,151,152], obesogenic chronodisruption [153], and the other specific traits associated with cardiometabolic pathologies typical of aging and prevalent among severely ill COVID-19 patients [134]. 

Chronic metabolic disease and cardiovascular and cerebrovascular disorders have reached an epidemic dimension in our era as an accelerated aging spread in industrialized countries and the hypokinetic–hypercaloric/lipidomic western lifestyle has become hegemonic in much of the world. The unfortunate connection between the pathological phenotype of these predisposing conditions and the increased vulnerability of such a high number of people to the most detrimental outcomes of COVID-19 is determinant in the colossal health threat, unprecedented in recent history, posed by this pandemic. Melatonin may come to the rescue of this dire situation, ameliorating the pathological coordinates of primary predisposing comorbidities and thereby providing countermeasures for the avoidance of highly diseased COVID-19 patients. Based on this background, supplementation of the internal pool of the powerful antioxidant, anti-inflammatory, and immunostimulant melatonin are a low-cost, low-toxicity and, more importantly, potentially efficient strategy to strengthen homeostasis mechanisms, achieve so-called “healthy aging” [134], and overcome the array of cardiometabolic complications that negatively impact the prognosis of COVID-19.

### 4.5. Melatonin and Inflammaging in the Context of COVID-19

The natural history of disease in modern Medicine includes an inflammatory component that is not merely epiphenomenal since the conceptualization of molecular pathogenesis gains consistency with the incorporation of cellular and signaling mechanisms of inflammatory response. Moreover, in many gnoseological entities such as sepsis or viral infections [154], destructive high-grade inflammation plays a leading role in the expression of pathological phenotype that is critical to control for ameliorating adverse prognosis. This is the case of COVID-19, in which the severe disease progression comes from a life-threatening immune–inflammatory cycle orchestrated by the overreaction of innate antimicrobial immunity and inflammation mechanisms activated by the presence of viral proteins [155]. The epidemiological picture of the current pandemic shows that people highly susceptible to infection and severe prognosis share a turn-on immune system and chronic inflammation [156], as people with primary comorbidities and/or frail immunocompromised elderly people over 65, especially male [157], who account for ~¾ of seriously ill COVID-19 patients [123]. To such an extent, the inflammation is decisive for the outcome of COVID-19 [158]. Thus, the fatal prognosis of hyper-inflammation and target organs in COVID-19 depends on the immune overreaction rather than on pathogen-mediated damage [159]. Indeed, patients in early COVID-19 stages display innate immune up-regulation, lymphocytopenia, and elevated serum content of cytokines (mainly TNF-α, IL-6), chemokines, and other chemoattractants [160], along with reduced launch levels of antiviral type I and III interferons [161], globally leading to an auto-inflammatory loop.

Once SARS-CoV-2 penetrates lung tissue targeting the conserved membrane Angiotensin Converting Enzyme-2 (or ACE2) receptors [162], the renin–angiotensin–aldosterone system is gathered and the NLRP3 inflammasome and the highly inflammatory cell-death associated pyroptosis are activated [163]. Afterward, recruited phagocytes release local pro-inflammatory factors and initiate T- and B-cell-dependent immune responses. Hence, different subtypes of effector T-cells or regulatory activated CD8^+^ T-cells eliminate epithelial infected cells or secrete exaggerated amounts of pro-inflammatory cytokines and chemokines into the bloodstream. This storm of cytokines induces the apoptosis and necrosis of affected cells, the release of their cellular contents, vascular endothelial hyper-permeability, and ultimately new inflammation inputs, which, in the interim of approximately one week, will devastate cells and tissue until the complete disruption of respiratory physiology [164]. 

Based on the aforementioned precedents on COVID-19 pathogenesis, the blockade of excessive cytokine release by targeting some of its critical triggers would avoid the multiple organ damage/failure and reasonably palliate the adverse outcome of the disease, as well as contribute to implementing effective curative strategies. In this regard, melatonin stands out as one of the best-positioned intervention therapy options to return to equilibrium the critical gears of uncontrolled immune inflammation. Although the current investigation into direct protection of ACE2 by melatonin is inconclusive [165], the evidence of its anti-inflammatory capacity is indisputable. Noteworthily, melatonin modulates inflammation as a hormesis-like process, activating moderate pro-inflammatory mechanisms in the course of the homeostatic immune response to intruder pathogens and contrarily stopping inflammatory mechanisms in contexts of hyper-inflammation, attenuating circulating cytokines and pro-inflammatory effectors [106]. For this reason, melatonin may ameliorate the exacerbated host inflammatory response and thereby prevent the irreversible pulmonary fibrosis and chronic respiratory damage of severely ill COVID-19 patients [10,166], as the indoleamine has demonstrated in experimental studies [167,168]. This damped response rests on positive modulation of anti-inflammatory cytokines such as IL-10 and attenuation of pro-inflammatory ones, such as IL-1β, IL-6, IL-8, or TNF-α exerted by melatonin [169]. A similar rationale supports the use of melatonin to treat gut complications caused by hyper-inflammation and exacerbated innate immune response against SARS-CoV-2 [170]. The combined anti-inflammatory and immunomodulatory capabilities of pleiotropic melatonin endow it with an extraordinary potential to prevent the gastrointestinal complications associated with SARS-CoV-2 infection [170]. In addition, the reciprocal interaction between the gut microbiome and pulmonary physiology, termed the “gut–lung axis”, provides microbiota with a relevant role in the immune homeostasis and the deadly recurrent ARDS in COVID-19 [171]. Melatonin reduces gut membrane permeability and is active in the chronoregulation and maintenance of a healthy balanced gut microbiome [172]. A recent work has elucidated the immunomodulatory importance of melatonin, both pineal and systemic, in COVID-19 severity, mediated by a complex myriad of factors, including the alpha 7 nicotinic acetylcholine receptor from lung epithelial and immune cells or the gut microbiome-derived and epigenetic regulator butyrate [173]. A noticeable meta-analysis of the anti-inflammatory capacity of melatonin administration has recently confirmed, based on 13 studies and a global cohort of 749 people, the reduction of TNF-α and IL-6 levels [174], suggesting that it would have a similar effect on COVID-19 patients. However, the initiation of melatonin supplementation must be carefully addressed to ensure its anti-inflammatory potential [31]. However it is hypothesized that the anti-inflammatory activity of melatonin is mainly achieved by orchestrating the suppression of toll-like receptor (TLR) signaling [175], the inhibition of the multiprotein platform inflammasome promoter NLRP3 (Nucleotide-binding Oligomerization Domain (NOD)-Like Receptor Pyrin domain containing 3) [176], and the blockage of the nuclear translocation of the NF-κB [76]. The result of binding viral proteins and/or genomic ssRNA by TLR/PAMPs would be the recruitment of the receptor–ligand complexes and the activation of apoptosis, lysosomal autophagy, and inflammasome-induced pyroptosis for the clearance of the virus [166]. Additionally, self-components from the autolytic processes activated, and particularly those released from stressed suboptimal mitochondria, initiate another immune–inflammatory cascade known as the “secondary cytokine storm”. These concatenated massive cytokine releases give rise to the fearsome continuous immune–inflammatory process that causes the extensive tissue and organ damage of the critical COVID-19 phase. In this scenario, melatonin may presumably prevent the secondary cytokine storm and associated hyper-inflammation of COVID-19. Different disease models have shown melatonin repressing pro-oxidant TLR2- and TLR4-mediated signaling cascades in the inflammatory phenotypes of ovarian cancer and coronary artery disease [175]. Moreover, TLR4 is commonly associated with the oxidative stress-sensitive NK-κB pathway and the triggering of inflammation. Therefore, in the context of COVID-19, the potential involvement of melatonin in the control of hyper-inflammation through the genetic and/or mechanistic inhibition of certain pro-oxidant TLRs is extremely consistent, as reported in some viral processes. Following these precedents and regarding COVID-19 moderation, in silico-based force-fields, the modeling of putative SARS-CoV-2 mRNA-TLR binding interactions has shed light on the probable activation of the TLR-dependent downstream inflammatory pathway by several mRNAs of SARS-CoV-2 [177], opening the way to melatonin intervention.

Melatonin also has the ability to prevent the activation of NLRP3 inflammasome in respiratory disease and other disturbances. This is the case of acute lung injuries such as sepsis or pneumonia, in which melatonin has been reported to reduce lung inflammation by selectively inhibiting the assembly of the NLRP3 inflammasome [178,179]. Excessive inflammation is a clue factor in the negative evolution of COVID-19 and other viral infections. Accordingly, it would make perfect sense that the NLRP3 complex directly or indirectly drives the hyper-inflammatory cascade associated with the explosive cytokine storm syndrome, as a large body of recent evidence points out [180]. Melatonin directly targets NLRP3 and precludes the activation of the inflammasome complex, enabling it to modulate inflammation. Therefore, it seems reasonable to launch the clinical trials with supplemental melatonin in the acute interval of ARDS, the height of hyper-inflammation of COVID-19, as recently proposed [25,31]. Nevertheless, direct experimentation is required on this matter to screen the auxiliary treatment of COVID-19 by the interference of inflammasome activation and open the alternative intervention routes and treatment schedules that are so urgently needed. 

In addition to the above findings, a hypothetical method that adds to the therapeutic possibilities of melatonin to moderate the inappropriate inflammatory response of COVID-19 is the control of Nuclear Factor (erythroid-derived 2)-like 2 (NRf2), a pivotal regulator of antioxidant responses that induce phase-II antioxidant enzymes [29]. Nrf2 is in a close mechanistic relationship with sirtuin 1 and NK-κB, whose close coordination in the protection from acute lung disease and acute respiratory distress syndrome has been raised as possible [181]. On a related topic, an association is observed between the cytokine release syndrome and the elevation of the gene expression modulator miR155, which also appears to be raised in the high-risk comorbidities of SARS-CoV-2 [106]. miR155 sustains the upregulation of cytokines in immunocytes and, in this regard, the demonstrated ability of melatonin to drop its levels [182] highlights the potential of this indoleamine in improving the clinical expression of COVID-19. Furthermore, the close relationship between melatonin and miR155 in different cell types is an additional indication that melatonin can be more efficient in the control of this transcriptional regulator than specific miR155-targeted drugs [106]. These studies provide strong support that exogenous and/or endogenous melatonin acts on inflammatory cascades and may correct the pro- vs. anti-inflammatory inputs in the course of mitigating hyper-inflammation. There is therefore enough evidence to take very seriously the adequacy of the functional biology of melatonin to the pathophysiology of COVID-19.

In parallel with activation of phagocytes and intracellular PRR/TLR pathways of innate immunity, melatonin cross-activates proliferation and the maturation of natural killer cells as well as T and B lymphocytes in the bone marrow and peripheral tissues [166]. These actions could help to deploy the adaptive cell/humoral immune response through specific antibodies fitted to the idiosyncrasy of SARS-CoV-2 and improve the detrimental outcome of COVID-19 patients. A proof-of-concept of the immune–endocrine axis and the role of melatonin in bidirectional communications is the production of melatonin in immune cells [183] and the association of inflammatory tone with nocturnal melatonin depletion [184]. The reinforcement of T-cells is relevant to the role attributed to melatonin against COVID-19 because in many asymptomatic patients or with mild symptoms, a low level of humoral antiviral response has been detected and, in contrast, a strong response mediated by T lymphocytes [45]. 

Melatonin has also revealed beneficial for shielding oxidative stress and inflammation generated by an excess of reactive oxygen and nitrogen species (RONS) in acute lung parenchyma injury and ARDS, as well as in the respiratory stress and delirium arising from intubation and assisted ventilation of patients under intensive care [185]. Particularly, circulating IL-6, the central milestone of the cytokine storm, is an independent predictor of lung injury and the severity of pneumonia in COVID-19 patients [158]. Critical COVID-19 patients present advanced inflammation, dyspnea, severe septic hypoxemia, and pneumonia that reduce breathing and respiratory efficiency and lead patients to long periods of intensive care and eventually intubation. The high pressure of oxygen in forced mechanical ventilation conditions produces oxidative stress and an epithelial–mesenchymal transition leading to fibrosis, thus making very plausible the role that melatonin could play in the management of this clinical phenotype. In addition, melatonin protects alveolar surfactant from peroxidation by infiltrating neutrophils, thus preventing the obliteration of pulmonary ways [186] and thereby improving the gas exchange in seriously diseased COVID-19 patients.

### 4.6. Mitochondrial Disruption Aggravating COVID-19

Mitochondrial dysfunction, such as depletion of energy generation (OXPHOS deficiency), RONS overproduction, or inhibition of membrane potential (membrane leakage), among others, is increasingly associated with the molecular pathogenesis of a growing number of diseases, including those neural, muscle, and endocrinopathies severely incapacitating and specifically denominated “mitochondrial disorders” [187]. Many viral infections may affect mitochondrial architecture and dynamics and interfere, either inducing or inhibiting their important spectrum of biochemical functions [188,189]. However, regarding COVID-19 pathogenesis, mitochondria have not focused enough interest, in spite of the fact that perturbation of this integrative center of energy and metabolism hinders an adequate host–pathogen defense [190]. It must be taken into account that immune response has a strong demand for biosynthetic and mitotic activities, which are strongly dependent on energy produced by mitochondria. In connection with immune interference, acute COVID-19 patients present subsets of dysfunctional T-cells with dysmorphic mitochondria exhibiting altered ultrastructure and cytochrome c release [191]. In agreement with this rule, recent studies of the global SARS-CoV-2 interactome map reported high-confidence protein–protein interactions that demonstrated the putative targeting of host mitochondria proteins by viral components [192,193], including the innate immune system. Similarly, computational machine learning models have predicted the preferential enrichment of genomic SARS-CoV-2 RNA in the mitochondrial matrix and nucleolus of the host [194]. Overall, SARS-CoV-2 may target mitochondria and disrupt their internal organization and functionality [195], so energy failure and increased RONS generation in the suboptimal stressed mitochondria determine the hyper-inflammation that aggravates the COVID-19 outcome [196]. With regard to this, the analysis of public transcriptome datasets has led to finding a transcriptional signature induced by SARS-CoV-2 that includes the induction of OXPHOS genes [197]. Analogously, transcriptome profiling of human lung cancer cells infected with SARS-CoV-2, influenza A virus, or MERS-CoV (subsequently validated on human nasopharyngeal specimens positive for COVID-19 and their control counterparts) revealed that SARS-CoV-2 can specifically disrupt inflammatory responses and mitophagy/autophagy machinery, as well as deregulate a myriad of genes linked to inflammation and cytokine signaling, cell cycle, RONS balance, mitochondrial organization, and translation [198]. These proof-of-concept studies demonstrate that “mitochondrial hijacking” to hinder the innate immune mechanisms and favor self-destructive hyper-inflammation and sepsis is strategic for viral infection [190]. Furthermore, the worse outcome and death risk of COVID-19 in males [199] has been related, among other unanswered hypotheses, to the sexual hormone-dependent dimorphism of mitochondria [200] and to sex-linked differences in immune response due to their matrilineal inheritance [201]. Of note, the apparent superiority in stress resilience of maternal mitochondria has been related to their greater performance in the production of melatonin [166]. 

Viruses evolved strategies to hijack mitochondrial machinery and evade the recognition by the viral sensor (RIG-I)-like receptors (RLRs). Once entering the cell, key intracellular receptors housed in the mitochondrial outer membrane named MAVS signalosome [202] usually recognize viral antigens and activate the defense system to restrain microbe invasion. In the case of SARS-CoV-2, it has been hypothesized that viral protein ORF9b would suppress MAVS downstream signaling (through Tumor necrosis actor Receptor-Associated Factor (TRAF)3 and TRAF6) and thereby the innate immunity and the related release of interferons could lose robustness [190]. In accordance with this prediction, it has been recently demonstrated that ORF9b from SARS-CoV-2 colocalizes on mitochondria and interacts with TOM70 protein in the outer membrane to suppresses IFN-I responses [203], and ORF9c interacts with complex I of the respiratory chain [204]. ORF9b may promote virus replication by inhibiting the apoptotic destruction of infected cells and by supporting their survival and viability through induction of mitochondrial elongation and fusion. In short, ORF9b disarticulates mitochondrial processes, such as mitophagy, which increases the oxidative stress, allowing inflammation and the mitochondrial complement to be kept fully functional [195]. Moreover, the hijacking of mitochondria deteriorates their structure, increases their permeability and the release of mitochondrial materials to the cytosol, and contributes to inflammation. In addition, SARS-CoV-2 depends on mitochondria (as well as endoplasmic reticulum and Golgi apparatus) to ambush itself, assemble the membrane-associated replication complexes, and put the genetic and biochemical machinery of the organelle at the service of its own replicative and immune escape requirements [194]. 

Recent findings have shown that melatonin is abundant and may be enzymatically synthesized in mitochondria [205,206,207]. Moreover, cellular and mitochondrial membranes are embedding melatonin transporters that allow bidirectional mobilization and concentration of the indoleamine [208]. Therefore, mitochondria can take extracellular melatonin when circulating or cerebrospinal fluid levels rise above normal [209]. All this evidence points out that this “second pool” of the indoleamine [165] is abundant because high levels are needed for the homeostasis of mitochondria. SARS-CoV-2 can dramatically manipulate the mitochondrial biochemistry, shifting their energy profile from OXPHOS to an abnormal preponderance of aerobic glycolysis (“Warburg effect”). This change maximizes ATP production and provides immune cells with energy and resources to sustain their intense phagocytic activity and massive release of cytokines [210]. In this regard, night-time pineal melatonin is active, resetting the immune system and strengthening defense responses through OXPHOS optimization [210]. Therefore, the exogenous melatonin should revert the preponderance of cytosolic glycolysis induced by SARS-CoV-2 and promote the switching to OXPHOS. Supplemental melatonin may improve mitochondrial metabolism and provide the acetyl-CoA for the in situ synthesis of the indoleamine [146]. Consequently, targeting glycolysis and resetting the energy metabolism of mitochondria to healthy interval exogenous melatonin may help to reverse the impact of SARS-CoV-2, especially the cytokine storm syndromes and ulterior devastating immune–inflammatory exacerbation. In this manner, the most severely detrimental COVID-19 symptomatology would be relieved [146].

Mitochondria are active sources of melatonin and also producers of large amounts of RONS and other free radicals. Nevertheless, melatonin preserves the homeostasis of mitochondrial structure and functionality thanks to its competence in scavenging RONS excess (melatonin binds up to 10 free radicals per molecule; [211]), which protects them from apoptosis and enhances their anti-oxidative systems [143]. Viral respiratory processes heal with hyper-production of free radicals and nitro-oxidative stress, leading to inflammation and tissue damage [212], hence the interest in the direct and indirect scavenging activity of melatonin as well as in the therapeutic relevance of its anti-inflammatory properties [213]. RONS are microbiocidal in origin and therefore are buffered by mitochondrial antioxidant systems into the physiological range [214]. Conversely, RONS excess produces chemical insults on cell macromolecules and induces the highly inflammatory mitochondrial lytic pyroptosis. In this regard, the ability of melatonin to quench the overload of RONS and mitigate nitro-oxidative stress is another reason to be confident in its therapeutic potential against the COVID-19 pandemic, as repeatedly postulated [31], since the protection deployed on mitochondrial membrane potential, RONS homeostasis, and energy metabolism is effective to orchestrate an innate immune response in the intensity range more lethal for SARS-CoV-2.

Dysregulation of ion trafficking across mitochondrial channels is a major cause of pathophysiology, as occurs with iron in COVID-19 in which a clear correlation between serum hyperferritinemia and disease severity as well as between ferritin blood load and circulating IL-6 cytokine have been reported [57,215]. At this point, the action of melatonin in moderating cytokine secretion, preventing oxidative stress, and reducing the hyperferritinemia associated with hemodialyzed patients under inflammation [216] supports the dual proactive role of melatonin in COVID-19 as an anti-inflammatory and regulator of iron overload. 

Given the short history of SARS-CoV-2 in science, many important details of its biology remain unknown. Soon, these lags will be elucidated, and new approaches to reducing infectivity, neutralizing tropism, cancelling spread-out, and overcoming the health issue of COVID-19 will be available. The natural history of COVID-19 is still being written, but an undoubted fact emerges from the chapters already known: SARS-CoV-2 enhances self-replication, impairing mitochondrial biosystems until defective autophagy and mitophagy, the deterioration of proteostasis, the depletion of OXPHOS in favor of anaerobic glycolysis, the overproduction of RONS, and the subsequent nitro-oxidative stress. All these impairments ultimately damage peripheral target organs such as the lungs, gut, or brain [217] and eventually lead to death. This central role played by mitochondria makes them strategic in the infective cycle of SARS-CoV-2 and therefore putting their biochemical capacities into play is one of the best options to treat COVID-19. Regarding this, mitochondria are active factories of melatonin production and one of the main operational centers of the indoleamine. In view of this, it seems clear that this molecule is a leading actor in the orchestration of immune, metabolic, oxidative, and inflammatory responses driven and coordinated by mitochondria.

### 4.7. Melatonin in Adjuvant Therapy Combination Against SARS-CoV-2 Infection

Evidence in the melatonin field has suggested its use as a combination treatment with the possibility of enhancing the therapeutic activity of different drugs and/or reducing the possible side effects when they are administered, which would suggest interesting beneficial perspectives. Additionally, melatonin has vasodilation effects on pulmonary arteries based on its antioxidant and anti-inflammatory capacities, and this vasoactive potential is another favorable support for adjuvant prescription of indoleamine to pandemic-affected individuals [218]. In this sense, adjuvant melatonin administration in combination with the current low-efficacy standard antiviral treatments promotes important improvements in symptomatology, clinical outcome, and support requirements of COVID-19 patients [113,219], in part because different molecules have different targets and mechanisms of action. Thus, melatonin can reconcile the need for delivering mechanical respiratory support in some acute COVID-19 patients and the damage inherent in forced ventilation. In accordance with its affordability and expected accuracy, much scientific press is echoing the formidable therapeutic and/or prophylactic expectations of adjuvant melatonin for the treatment of COVID-19 patients, alone or in synergistic combination with other natural products that share its nuclear receptor and signaling profile, such as vitamin D [220]. More recently, it has been suggested that the supplementation of vitamin D, zinc, melatonin, and possibly additional nutraceuticals could reduce the risk and aid control of COVID-19 and a range of other viral infections [221]. Therefore, in the context of “antioxidant therapy”, a cocktail with antioxidant supplements such as vitamins C and E, *N*-acetylcysteine, and melatonin in combination with the hemorrheologic agent pentoxifylline could contribute to the mitigation of the aggressive and lethal development COVID-19 [222]. Likewise, given the rich evidence that this extensive literature compiles and the international prestige of some of the personalities that bona fide endorse this clinical strategy, it is difficult for us to find resounding words and a new convincing voice to persuade public health dealers and professionals about the urgency of undertaking standardized clinical trials. Additionally, melatonin upregulates B-cell proliferation and therefore potentiates refined humoral immune responses [166] and modulates positively the innate and adaptive immunities as a vaccine adjuvant [223]. Noticeably, the immunomodulatory action as adjuvant of melatonin may enhance the effectivity of vaccination in immunocompromised individuals, such as aged people and patients with comorbidities, who are at great risk of lethal COVID-19. Specifically, melatonin pre-treatment can increase the intensity and temporal coverage of the immune response, enhancing natural killer and CD4^+^ cells in addition to reducing the side effects of vaccination [23,32]. Melatonin has also been documented targeting the activity of CD147, which takes part in the cytokine storm that causes inflammatory injury in the lung [101]. In this context, and considering the involvement of CD147 S glycoprotein in RONS production and inflammatory responses, supplemental melatonin has been proposed as a possible adjuvant to ameliorate the COVID-19 symptomatology and the side-effects associated with the current repurposed therapeutics, especially among frail, elderly, and immune-compromised patients [104]. Furthermore, using a systems biology and artificial-intelligence-based approach to reducing the severe pulmonary complications caused by SARS-CoV-2, the combined action mechanism of melatonin and pirfenidone predicted that they may modulate the high levels of proinflammatory chemokines and cytokines, improving the pathophysiology of COVID-19 patients [224,225]. In this same line, using a network-based methodology for systematic identification of putative repurposable drugs was identified as a combination of mercaptopurine plus melatonin, which may synergistically inhibit multiple cellular targets in the infectious process of SARS-CoV-2 [24].

Consequently, in the pressing context created by the COVID-19, we herein propose that melatonin upregulation or its adjuvant administration with current repurposed pharmacological prescriptions can be pivotal to achieving more effective treatments to curb the current spread of disease and improve the clinical management of patients. As other authors have emphasized, in the worst scenario, “melatonin is not yet guaranteed as an effective treatment, it likely would be useful and is unlikely to do any harm” [165], and we will have nothing to regret. On the contrary, in the probable case that it provides part of therapeutic expectations demonstrated in other pathological scenarios, the contribution of ancient melatonin to global public health in this dramatic sanitary alert will be historical.

This preliminary evidence points out the prophylactic and/or supportive therapeutic potential of adjuvant melatonin in respiratory and non-respiratory complications of COVID-19. Specifically, self-limitation or abrogation of the local and systemic inflammatory mechanisms account for the multiply reported benefits of melatonin in respiratory disorders with pulmonary involvement [226], and it could be analogously assumed against the lung damage in COVID-19 patients. The initial backgrounds, therefore, highlight the rationale for undertaking with no delay observational studies of large-scale cohorts, as well as randomized clinical trials, to validate the clinical effectivity of the indoleamine in reducing symptoms and/or its prophylactic utility [25,29]. In the absence of effective drugs against SARS-CoV-2 and palliative therapies for COVID-19 and taking into consideration the difficulties encountered by vaccination against previous viral processes [18], it is time to implement other therapy options. In this regard, the combination of current treatment protocols with multitasking melatonin may be the gold standard that humanity imperatively needs to fight COVID-19 and that science is desperately searching for.

The therapeutic potential of melatonin to fight COVID-19 is very wide, as we have depicted along point 4. To summarize the properties, we elaborate in Table 2. 

## 5. Melatonin: From Regulatory to Safe and Effective Interventions in COVID-19

In the European Union (UE), the European Medicines Agency (EMA) melatonin first approved Circadin, 2 mg of melatonin, for medical use in adults over 55 to treat insomnia in 2007 (EMA/273802/2010); to date, two registered medicines contain melatonin as an active substance, Circadin and Slenyto (EMA/570422/2018). Circadin contains 2 mg melatonin tablets, and it needs a prescription. Slenyto has been authorized for treating insomnia in children and adolescents (from 2 to 18 years old) who have autism spectrum disorder or Smith-Magenis syndrome (SMS) (a condition that can lead to learning difficulties). While the recommended dose is 2 mg, it is available as tablets from 1 to 5 mg. This treatment should be supervised at least every six months and only if it is beneficial. Nonetheless, melatonin is designated as an orphan medicinal product for six rare diseases: treatment of perinatal asphyxia, SMS, necrotizing enterocolitis, neonatal sepsis, neonatal encephalopathy, and acute radiation syndrome. The Orphan Regulation (Regulation (EC) No 141/2000) lays down the European Union procedure for the designation of orphan medicines; it defines the development and placing onto the market designated orphan medicines and establishes the Committee for Orphan Medicinal Products (COMP). Orphan products are still under investigation and are considered for orphan designation based on their potential activity. An orphan designation is not a marketing authorization. Therefore, it is necessary to demonstrate quality, safety, and efficacy before it can be granted a marketing authorization.

Depending on whether melatonin is treated as a drug (Directive 2001/83/EC) or as a dietary supplement (Regulation (EC) No 1924/2006), authorization and quality requirements differ significantly. The EU legal framework for human medicines guarantees higher standards of quality and safety. In other countries, such as the US or Canada, melatonin is an over-the-counter medication, up to a dose of 10 mg, without a prescription, and it is not regulated as strictly as other medicines. The FDA classifies melatonin as a dietary supplement; thus, there is no official FDA approval.

It is important to highlight that in the technical data of the UE-authorized melatonin drug, no severe adverse effects had been observed even at high doses (300 mg). Indeed, the incidence of adverse effects in treated patients is nearly the same as in the placebo group (EPAR for Circadin. Procedure No. EMEA/H/C/695). Based on this frame, the administration of low doses of melatonin may be effective for mild disorders such as insomnia, but when it comes to more severe diseases and in light of the previous doses administered in human clinical trials with effective results, it is likely that higher doses are needed.

## 6. Conclusions

Although melatonin does not have virucidal capacity per se, supplemental melatonin interceded favorably, eradicating the pathogenicity of highly deadly viruses [16,19,227], as widely documented by numerous reports of different casuistry, among which the prevention of the hemorrhagic shock syndrome from Ebola virus infection is especially encouraging [17]. In this issue, taking into account the pharmacological profile of melatonin, which highlights formidable homeostatic abilities to moderate inflammation, mitigate redox stress, and buffer immune response, this indoleamine should occupy a preferential place among the molecules to be tested with regard to slowing down the hyper-inflammation of COVID-19 patients and increasing the success of their clinical management. 

Over the last few months, immense efforts have been made to reposition existing drugs and alleviate the most threatening complications of the disease. So far, none of the explored possibilities have yielded widely favorable results; new possibilities continue to be investigated. Fortunately, the sudden development of dozens of vaccines, whose international distribution has recently begun, has been more successful. However, the recurrent appearance of genetic variants of SARS-CoV-2 raises the suspicion that the current pandemic will lead to future seasonal epidemic waves, in the style of those that occur every year. The need for palliative treatments against COVID-19 will not disappear despite the worldwide extension of vaccination campaigns, and therefore the importance of melatonin is not restricted to the control of the current pandemic but is projected to the clinical management of the disease in the future. However, a recent systematic review has reported twenty-four studies with slight adverse events attributed to melatonin (sleepiness, headache, or fatigue), while other more serious side effects were seen at very high doses or in specific populations [68]. In this context, some reports have evidenced that short-term melatonin can sometimes lead to opposite effects to those expected, as a probable cause of diarrhea [228] or gynecomastia [229] or as a trigger of Crohn´s disease [230]. Being a natural molecule, extensively studied, with high availability and tolerability, safe and low priced, the cost/benefit ratio is extremely advantageous for both purposes. It is therefore imperative to structure an international program of clinical trials, low-cost but with an immense potential benefit of historical dimensions. In a time in which vaccines are beginning to be distributed in many industrialized countries, some health international organizations denounce the gap of access in poor communities, claiming for a fair distribution of the unique measure that, in the medium- and long-term, can stop the ongoing challenge of the COVID-19 pandemic and save millions of lives. In this regard, we envisage that melatonin can be an immediate, effective, and global palliative. Therefore, in our opinion, the study of the clinical possibilities of supplemental melatonin in the management of COVID-19 is not simply another option to be tested, it is an ethical duty of translational research in clinical therapeutics. Our wish is that the current update of the possibilities of melatonin to impede SARS-CoV-2 morbimortality motivates immediate research programs that, as soon as possible, put into the hands of the clinicians a successful strategy for the clinical management of the health emergency of the COVID-19 pandemic that came by surprise into our lives just over a year ago.

## Figures and Tables

**Table 1:** Ongoing clinical trials with melatonin to treat SARS-CoV-2 infection.

	Cochrane Library ID Number:	Route Dose/Rationale Treatment Duration	Participants	Blinding/ Comparison Groups	Primary Outcomes	Secondary Outcomes
1	CN-02137224	Oral	30 participants with COVID-19 symptoms	Quadruple * Placebo	Incidence of serious adverse effects Discontinuation secondary to toxicity	Hospitalization COVID-19 related symptoms Rate of resolution of COVID-19 related symptoms Mortality
		10 mg/3 times day				
		14 days				
2	CN-02148200	Oral	390 participants with mild COVID-19	Quadruple * Toremifene + Melatonin Melatonin + Placebo Placebo	The peak increase in COVID-19, sign and symptom score	Nadir Oxygen Saturation Peak Heart Rate Time to COVID-19 Sign and Symptom score resolution Time to WHO 7-point ordinal scale score of 3 or higher #
		2 times/day (Days 1, 2) 100 mg (40 mg/60 mg) (Days 3–14) 60 mg (20 mg/40 mg)				
		14 days				
3	CN-02187811	Oral	Participants with confirmed COVID-19	Not shown Placebo	Body temperature Oxygen saturation Respiratory rate	C-reactive protein. Incidence of serious adverse events Lymphocytopenia
		50 mg/day				
		7 days				
4	CN-02188224	Oral	Participants mild to moderate COVID-19	Single blinded Not placebo	Headache, lung infection CRP (C reactive protein) fever, vomiting, no smelling, no taste, cough, respiratory distress	-
		6 mg/day				
		14 days				
5	CN-02168227	Oral	Participants with confirmed COVID-19	Double *** Placebo	SARS-CoV-2 infection rate	Frequency of respiratory tract infections Change from baseline immune cells and markers Treatment-related adverse events
		3 mg/day				
		8 weeks				
6	CN-02148187	Oral	150 participants with confirmed COVID-19	Triple ** Placebo	Symptom severity	Symptom progression
		10 mg/day				
		14 days				
7	CN-02174361	Intravenous	18 participants with confirmed COVID-19: ICU critically ill adults with acute hypoxemic respiratory failure	Quadruple * Placebo	Mortality	-
		5 mg/Kg b.w./ day/ 6 h (maximum daily dose 500 mg).				
		7 days				
8	CN-02187792	Oral	Hospitalized participants with confirmed COVID-19	Not shown No placebo	Need to oxygen therapy rate Rate of sleep and depression ICU time	-
		36 mg (18mg/12h)				
		7 days				
9	CN-02103276	Oral	450 participants Healthcare workers not having a previous COVID-19 diagnosis	Quadruple * Placebo	SARS-CoV-2 infection rate	-
		2 mg				
		12 weeks				
10	CN-02170332	Oral	Participants with confirmed COVID-19	Not blinded No placebo	Heart rate Number of breaths/min Course of the disease Rate of decline of lung infection	-
		40 mg (10 mg/6 h)				
		10 days				
11	CN-02195959	Oral	60 participants with confirmed COVID-19 and moderate pneumonia	Double *** Placebo	Recovery rate of clinical symptoms Oxygen saturation Improvement of serum inflammatory parameters; C-reactive protein, tumor TNF-α, IL-1β, and IL-6	-
		50 mg				
		7 days				

* Quadruple = Participant, Care Provider, Investigator, Outcomes Assessor; ** Triple = (Participant, Care Provider, Investigator); *** Double binded = Participant and Investigator Blinded; - = Data not shown; b.w. = body weight # WHO 7-point ordinal scale: a.—not hospitalized, no limitation of activities (or resumption of normal activity); b.—not hospitalized but a limitation on activities; c.—hospitalized, not requiring supplemental oxygen; d.—hospitalized, requiring supplemental oxygen (low-flow, e.g., nasal prong); e.—hospitalized, requiring non-invasive ventilation and/or high-flow oxygen; f.—hospitalized, on invasive ventilation or ECMO; g.—death

**Table 2:** Melatonin potential effects against COVID-19 disease.

COVID-19 Actions	Melatonin Potential Properties
Sleep and circadian rhythms dysregulation. Melatonin deficiency = higher risk	Resynchronization of circadian disruption [10,87,91].
Refractory hypoxemia and myocardial injury. Thrombotic events and inflammatory injury in the lung and heart failure.	Binds viral protease Mpro [27]. Exerts anti-PAK1 activity [114].
Low-grade basal inflammation, weakening of immune and antioxidant defenses, and metabolic syndrome abnormalities are predisposing conditions to COVID-19 aggravation.	Controls the innate and adaptive immunity [140] and inflammation [141]. Regulates neuroimmune–endocrine system [142]. Reduces oxidative stress and contributes to preventing hyper-inflammation and innate immune exacerbation [143,144,145].
Obesity, cardiac disorders, and type 2 diabetes increase mortality.	Melatonin-related signaling pathways have an extensive influence on glucose homeostasis and energy metabolism [147]. Improves diabetes and metabolic syndrome [148,149,150,151,152], obesogenic chronodisruption [153], and other traits associated with cardiometabolic pathologies [131,134].
Life-threatening immune–inflammatory cycle.	May ameliorate the exacerbated host inflammatory response [10,18,25,31,169,186].
Mitochondrial disruption.	May improve mitochondrial metabolism and reset energy metabolism [31,146,210]
